# Mutant-Based Model of Two Independent Pathways for Carotenoid-Mediated Chloroplast Biogenesis in Arabidopsis Embryos

**DOI:** 10.3389/fpls.2019.01034

**Published:** 2019-08-27

**Authors:** Eva Vranová, Diana Kopcsayová, Ján Košuth, Maite Colinas

**Affiliations:** ^1^Institute of Biology and Ecology, Pavol Jozef Šafárik University in Košice, Košice, Slovakia; ^2^VIB-UGent Center for Plant Systems Biology, Ghent, Belgium

**Keywords:** isoprenoids, *Arabidopsis thaliana*, carotenoids, chloroplast biogenesis, mutants

## Abstract

Chloroplasts are essential for autonomous plant growth, and their biogenesis is a complex process requiring both plastid and nuclear genome. One of the essential factors required for chloroplast biogenesis are carotenoids. Carotenoids are synthesized in plastids, and it was shown that plastid localized methylerythritol 4-phosphate (MEP) pathway provides substrates for their biosynthesis. Here, we propose a model, using results of our own mutant analysis combined with the results of others, that a MEP-independent pathway, likely a mevalonate (MVA)-dependent pathway, provides intermediates for chloroplast biogenesis in Arabidopsis embryos. The pattern of this chloroplast biogenesis differs from the MEP-dependent chloroplast biogenesis. In MEP-dependent chloroplast biogenesis, chloroplasts are formed rather uniformly in the whole embryo, with stronger chlorophyll accumulation in cotyledons. In a MEP-independent pathway, chloroplasts are formed predominantly in the hypocotyl and in the embryonic root. We also show that this pattern of chlorophyll accumulation is common to MEP pathway mutants as well as to the mutant lacking geranylgeranyl diphosphate synthase 11 (GGPPS11) activity in plastids but expressing it in the cytosol (GGPPS11cyt). It was recently described that shorter GGPPS11 transcripts are present in Arabidopsis, and they can be translated into active cytosolic proteins. We therefore propose that the MEP-independent pathway for chloroplast biogenesis in Arabidopsis embryos is an MVA pathway that provides substrates for the synthesis of GGPP *via* GGPPS11cyt and this is then transported to plastids, where it is used for carotenoid biosynthesis and subsequently for chloroplast biogenesis mainly in the hypocotyl and in the embryonic root.

## Introduction

Chloroplasts are essential for plants. They are responsible for CO_2_ fixation, biosynthesis of carbon skeletons, fatty acids, amino acids, pigments, hormones, vitamins, and other isoprenoid metabolites. Generally, the chloroplast develops from the undeveloped proplastid, which contain vesicles, but no differentiated structures. During this differentiation, thylakoids are formed and stacked into defined grana (Pogson et al., 2015). *Arabidopsis thaliana* belongs to the group of plants in which photosynthetically active chloroplasts are formed already during embryogenesis and are therefore referred to as chloroembryos (Puthur et al., 2013; Allorent et al., 2015). Chloroplasts are in Arabidopsis embryos formed at the globular stage, when they are seen as nonrandomly distributed chloroplast-containing cells (Tejos et al., 2010). In the heart stage of embryo development, chloroplasts are detected in epidermal cells as well as in the central region of the embryo. Torpedo stage embryos have chloroplast-containing epidermal cells and a central band of chloroplast-containing cells in the cortex layer, just below the shoot apical meristem. In the walking-stick stage of embryogenesis, chloroplasts are present in the epidermal and cortex cells of the cotyledons and hypocotyl and in the endodermal and cortex cell layers of the embryonic root. Chloroplasts appear reduced or absent from the provascular tissues and from the columella. These results suggest that there is a tight regulation of plastid differentiation during embryogenesis that generates specific patterns of chloroplast-containing cells in specific cell layers at specific stages of embryogenesis (Tejos et al., 2010). In a subsequent desiccation phase, these chloroplasts de-differentiate into non-photosynthetic, colorless leucoplasts, called eoplasts (Mansfield and Briarty, 1991; Mansfield and Briarty, 1992).

### Carotenoids and Chloroplast Biogenesis

Chloroplast biogenesis is a complex process that is controlled by both nuclear and plastidial genomes (Pogson et al., 2015). One of the factors that contribute to the chloroplast biogenesis is carotenoids, which, by itself, are the components of the photosynthetic apparatus. It has been demonstrated that plastids in mutants in the early steps of the carotenoid biosynthetic pathway (*pds3, zds/spc1-2/clb5, hst/pds2/clb2*, **Figure S1**) cannot transit from the proplastid to the chloroplast stage (Gutiérrez-Nava et al., 2004; Dong et al., 2007; Tian et al., 2007; Qin et al., 2007; Chao et al., 2014). Similarly, the transition of proplastid to etioplast, a plastidial form that is present in dark-grown seedlings, is dependent on the functional conversion of phytoene to carotenoids by carotenoid isomerase CRTISO (**Figure S1**, Park et al., 2002). The essential role of carotenoids in plastid biogenesis is further strengthened by the fact that all types of plastids, except proplastids, can synthesize carotenoids (Li et al., 2016).

### Mutants as a Tool to Study Chloroplast Biogenesis

In the screen for the chloroplast biogenesis (*clb*) mutants that are albinos, five mutants that belong to either the methylerythritol 4-phosphate (MEP) pathway, which synthesizes precursors for the carotenoid biosynthesis, or the carotenoid pathway were identified (Gutiérrez-Nava et al., 2004, **Figure S1**). *cla1*, *clb4*, and *clb6* mutant plants are affected in the expression of the deoxy-xylulose-5-phosphate synthase (DXS1), hydroxy-3-methylbut-2-enyl diphosphate synthase (HDS), and hydroxy-3-methylbut-2-enyl diphosphate reductase (HDR), respectively; these three enzymes each catalyze different steps in the MEP pathway (Gutiérrez-Nava et al., 2004; Guevara-García et al., 2005; de Luna-Valdez et al., 2014). *clb2* plants are affected in the expression of the homogentisate prenyltransferase (HST), active in the plastoquinone-9 biosynthetic pathway (Tian et al., 2007; Chao et al., 2014; de Luna-Valdez et al., 2014) and *clb5* plants in the expression of ZDS (ζ-carotene desaturase) enzyme (Dong et al., 2007; Avendaño-Vázquez et al., 2014; de Luna-Valdez et al., 2014), which is responsible for the biosynthesis of the essential carotenoid lycopene. Since plastoquinone is required as an intermediate electron carrier between carotenoid desaturases, similarly to ZDS, it is essential for the conversion of phytoene to lycopene. Interestingly, based on the plastid morphology, chloroplast development seems to be arrested earlier in *clb5* and *clb2*, and slightly later in *cla1*, *clb6*, and *clb4* mutants (Gutiérrez-Nava et al., 2004). Plastids of *clb2* and *clb5* lack appressed internal membranes and have large vesicle-like structures with unknown contents, similar to those found in proplastids. By contrast, the chloroplasts of *cla1, clb6,* and *clb4* contain linear appressed membranes. All mutants have none or severely reduced levels of chlorophylls and carotenoids and have an albino phenotype (Gutiérrez-Nava et al., 2004). In agreement with the more developed morphology of plastids in the MEP pathway mutants, they also have higher level of carotenoids and chlorophylls compared to carotenoid pathway mutants, and their embryos show chlorophyll fluorescence. While chlorophyll autofluorescence from the wild-type chloroplasts is clearly detected throughout the embryo, in the MEP pathway mutants, fluorescence is detected more in the hypocotyl region (Gutiérrez-Nava et al., 2004). Analysis of the additional MEP and carotenoid pathway mutants outside of this screen confirmed the similar phenotype for the respective group of mutants (Mandel et al., 1996; Estévez et al., 2000; Budziszewski et al., 2001; Guevara-García et al., 2005; Dong et al., 2007; Qin et al., 2007; Hsieh and Goodman, 2005, Hsieh and Goodman, 2006; Tian et al., 2007; Hsieh et al., 2008; Chao et al., 2014).

### GGPPS and Carotenoid Biosynthesis

**Figure 1:**
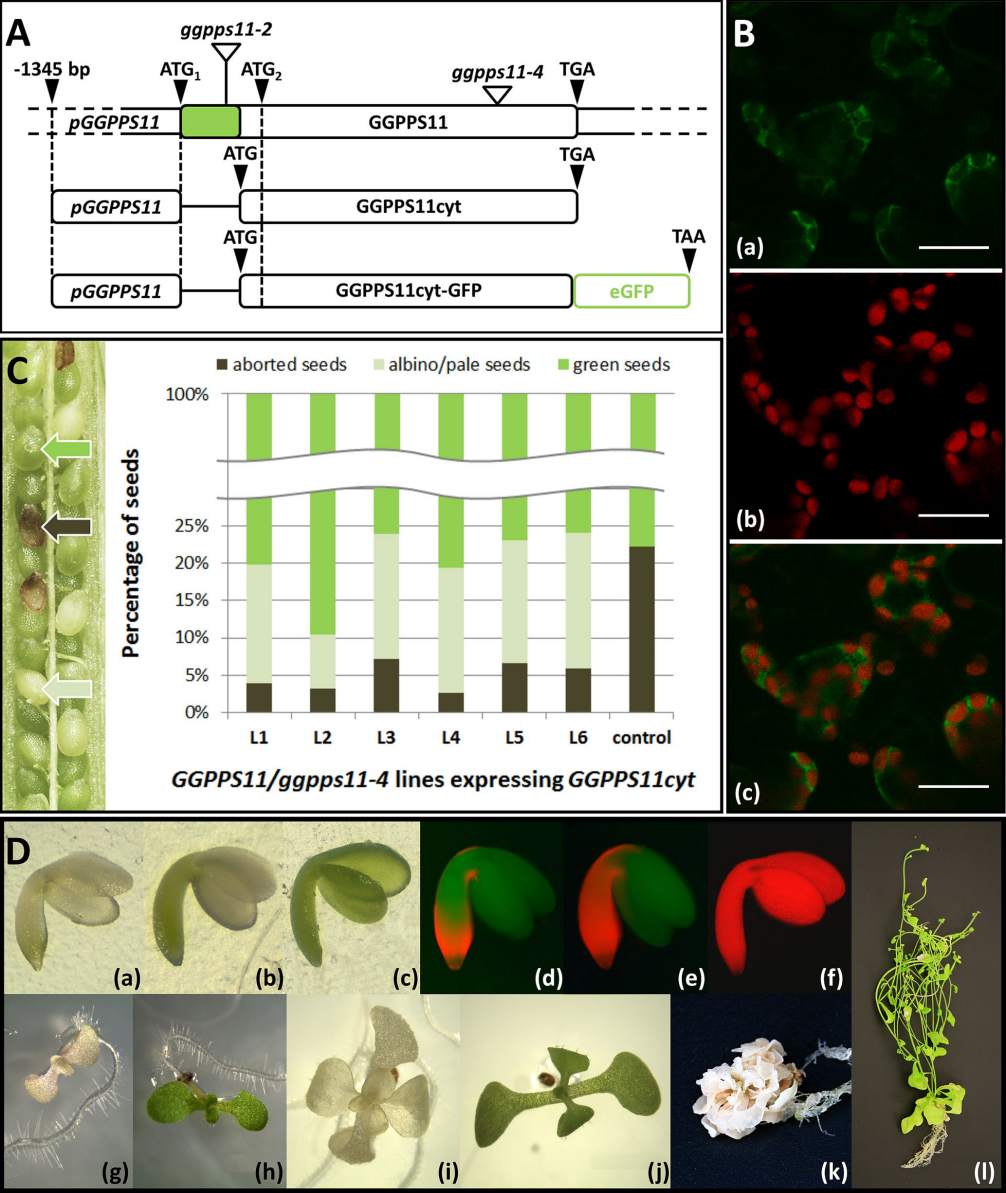
Complementation of *ggpps11-4* loss-of-function mutant by GGPPS11cyt. **(A)** Mutants and constructs mentioned or used in this work. The protein-coding sequence (which lacks introns), from which GGPPS11 encoding constructs are derived, is shown as a white box with a green section corresponding to the predicted plastid targeting sequence. The positions of translation initiation start and stop codons are shown as black triangles. The positions of T-DNA insertions are represented as white triangles. *pGGPPS11*::GGPPS11cyt and *pGGPPS11*::GGPPS11cyt-GFP are constructs encoding cytosol-targeted GGPPS11 with or without fused green fluorescence protein (eGFP). *GGPPS11* promoter (*pGGPPS11*, −1345 bp) is driving the expression. To clone *GGPPS11cyt*, GGPPS11 coding sequence was truncated by the length of the chloroplast transit peptide based on the TargetP prediction (http://www.cbs.dtu.dk/services/TargetP/; Nielsen et al., 1997; Emanuelsson et al., 2000). *GGPPS11cyt* was amplified from wild-type *A. thaliana* cDNA with primers G11cyt_fwd and G11cyt_rev (**Table S1**). *pGGPPPS11* was amplified from the wild-type *A. thaliana* gDNA with a pair of primers pG11_fwd and pG11_rev (**Table S1**). Cloning procedure is described in Supplementary Materials and Methods. **(B)** Subcellular localization of the GGPPS11cyt-eGFP fusion protein. Confocal microscopy of *A. thaliana* seedlings expressing *GGPPS11cyt-GFP* together with the red fluorescence of chlorophyll. The first row shows eGFP in green, the second shows fluorescence of the chlorophyll in red, and the third shows the overlay of the two channels. Bars represent 150 μm. Leaves of young seedlings were analyzed with an Olympus FV1000 confocal microscope (Olympus Corp.). The images were captured and analyzed in Olympus FluoView FV10-ASW (Olympus Corp.) eGFP was excited at 488 nm, and its emission signal was collected at 505–525 nm. Chlorophyll was excited at 543 nm, and its emission signal was collected at 655–755 nm. Transformation procedure is described in Supplementary Materials and Methods. **(C)** Partial rescue of *ggpps11-4* embryo lethal phenotype by GGPPS11cyt. Left: Picture of a silique of heterozygous *GGPPS11/ggpps11*-4 mutant ectopically expressing *pGGPPS11::GGPPS11cyt* gene. Green, aborted and albino/pale seeds were found. Right: Ratio of green, albino/pale and aborted seeds found in individual transformants. A total of 350–772 of seeds were counted from each plant. Percentage of albino/pale green seeds in siliques is as follows: 15.9% in L1, 7.3% in L2, 16.8% in L3, 16.8% in L4, 16.6% in L5, 18.3% in L6. Seeds were all analyzed visually using Leica EZ4 D stereo microscope and free software LAS EZ V3.2.1 (Leica Microsystems). Green, well-developed siliques were cut open and the T2 segregating seed population was analyzed. Transformation procedure is described in Supplementary Materials and Methods. **(D)** Partial complementation of *ggpps11-4* phenotype by GGPPS11cyt. Phenotypes of representative albino/pale green embryos (a–f), 9-day-old seedlings (g–h), 17-day-old plantlets (i and j), and 2-month-old plants (k and l) that developed from these embryos; (a, b, d, e, g, i, k) *ggpps11-4* mutant complemented with GGPPS11cyt; (c, f, h, j, l) wild type. Seedlings and plants were cultivated on MS plates supplemented with 1% sucrose, and in the case of mutants, phosphinotricin (PPT) 10 μg/ml and kanamycin (Kan) 25 μg/ml were added. Embryos, seedlings, and plantlets were all analyzed visually using a stereo microscope (Leica EZ4D) (a-c, g-j) or a fluorescence microscope (Olympus BX51) using an Olympus WIB filter cube to detect chlorophyll autofluorescence (d–f) or camera (Olympus E-520 camera and Quick Camera software) (k, l). Further processing of images was done in ImageJ (https://imagej.nih.gov/ij/).

The substrate for the carotenoid biosynthesis is geranylgeranyl diphosphate (GGPP), which is synthesized by GGPP synthase (GGPPS). GGPPS in *A. thaliana* is encoded by five isozymes localized in plastids (two), the endoplasmic reticulum (two), and mitochondria (one) (Beck et al., 2013; Nagel et al., 2015; Ruiz-Sola et al., 2016b). Only GGPPS11 that localizes to plastids contributes *in planta* significantly to the carotenoid biosynthesis (Ruiz-Sola et al., 2016a, Ruiz-Sola et al., 2016b). *ggpps11* loss-of-function mutant surprisingly does not show albino phenotype at the seedling stage, but arrests at the heart stage of development (Ruiz-Sola et al., 2016a; Ruiz-Sola et al., 2016b). Phenotype is therefore more severe than the phenotype of MEP pathway mutants or mutants in carotenoid biosynthetic pathway. Nevertheless, the *ggpps11-2* line (SALK_015098), which harbors a T-DNA insertion in the plastid targeting sequence of the GGPPS11 (**Figure 1A**), has a seedling-lethal albino phenotype, visually similar to other MEP pathway or carotenoid pathway mutants (Ruiz-Sola et al., 2016a). It was suggested that in this mutant, a second ATG that exists downstream of the T-DNA insertion could act as an alternative translation initiation site to generate a shorter protein. Transcripts of different length allowing translation of both plastidial and cytosolic protein are generated also in wild-type Arabidopsis plants, and it was proposed that both proteins may be synthesized in Arabidopsis. Analysis of total amount of carotenoids in the *ggpps11-2* albino mutant demonstrated that it has similar level of carotenoids as the *dxs-1* MEP pathway mutant, while in the same assay, almost no carotenoid level was detected in the carotenoid pathway *psy-1* mutant (Ruiz-Sola et al., 2016a). We were interested to see whether *ggpps11* loss-of-function mutant, which expresses GGPPS11 only in the cytosol, will phenocopy MEP pathway mutants regarding the chlorophyll fluorescence pattern in embryos.

### *ggpps11* GGPPS11cyt Embryos Phenocopy MEP Pathway Mutants

We have complemented *ggpps11-4* loss-of-function mutant line (SAIL_712_D06) (Ruiz-Sola et al., 2016b) with the *GGPPS11* cDNA lacking the chloroplast transit peptide sequence (*GGPPS11cyt*) driven by its own promoter *pGGPPS11* (**Figure 1A**). To verify localization of the GGPPS11cyt in the cytosol, the C-terminal fusion with the green fluorescent protein (GFP) was made and cytosolic localization of the GGPPS11cyt-GFP protein was confirmed (**Figure 1B**). On average, 22% of embryos in siliques of *GGPPS11/ggpps11-4* are developmentally arrested at the heart stage of embryogenesis (Ruiz-Sola et al., 2016a; Ruiz-Sola et al., 2016b). Approximately 16.5% (e.g. 75% of 22%) of embryos were expected to be partially complemented by the GGPPS11cyt and have albino or pale green phenotype (Ruiz-Sola et al., 2016b). As seen in **Figure 1C**, the number of partially complemented seeds, which are albino or pale green, is indeed approximately 16.5% in majority of lines. Nevertheless, in line L2, the number of albino/pale green seeds is much lower (7.3%), suggesting that some of the complemented embryos are green, similar to the wild type. Transgenic *ggpps11-4* GGPPS11cyt embryos are albino and show phenotype resembling that of MEP pathway loss-of-function mutants (*cla1*, *clb4*, and *clb6*) (Gutiérrez-Nava et al., 2004) (**Figure 1D**). Embryos have a weak but significant level of red chlorophyll autofluorescence localized in the hypocotyl, in the embryonic root, and in the region right under cotyledons. (**Figure 1D**, d,e). Chlorophyll in these embryos is also visible under normal light conditions (**Figure 1D**, a,b). We have also checked several MEP pathway mutants, including *dxs-1* and *dxr*, where a similar pattern of chlorophyll fluorescence was observed (Gutiérrez-Nava et al., 2004), under stereo microscope using normal light conditions (**Figure S2**). Chlorophyll is seen in these mutants in hypocotyl and in embryonic root as well. Thus, MEP pathway mutants and *ggpps11-4* mutant expressing GGPPS11 activity in the cytosol belong phenotypically to the same class of mutants. In addition to albino/pale green *ggpps11-4* GGPPS11cyt seeds, we have observed that in several *ggpps11-4* lines complemented with *pGGPPS11:GGPPS11cyt* construct, more green seeds, than expected, were observed (**Figure 1C**, L2). Embryos of those seeds were usually not completely green, but had more chlorophyll, also in cotyledons. The pattern of chlorophyll distribution was similar to that of albino embryos. Chlorophyll content was strongest in the embryonic root and in the hypocotyl and continued towards cotyledons (**Figure S3**). Seedlings and plants derived from those embryos were pale green, developed stems and flowers more often, and had rather variegated-like phenotype. Such phenotype was so far not observed in loss-of-function albino MEP pathway mutants (Mandel et al., 1996; Estévez et al., 2000; Budziszewski et al., 2001; Hsieh and Goodman, 2005, Guevara-García et al., 2005; Hsieh and Goodman, 2006; Hsieh et al., 2008; Avendaño-Vázquez et al., 2014). The reason for greener phenotype is currently unknown and we can only speculate about it. Since the phenotypic pattern varies between the complemented lines, it may be rather associated with the locus where GGPPS11cyt is inserted. This can influence expression of GGPPS11cyt and the amount of GGPP that is generated.

## Summary and Perspective

**Figure 2:**
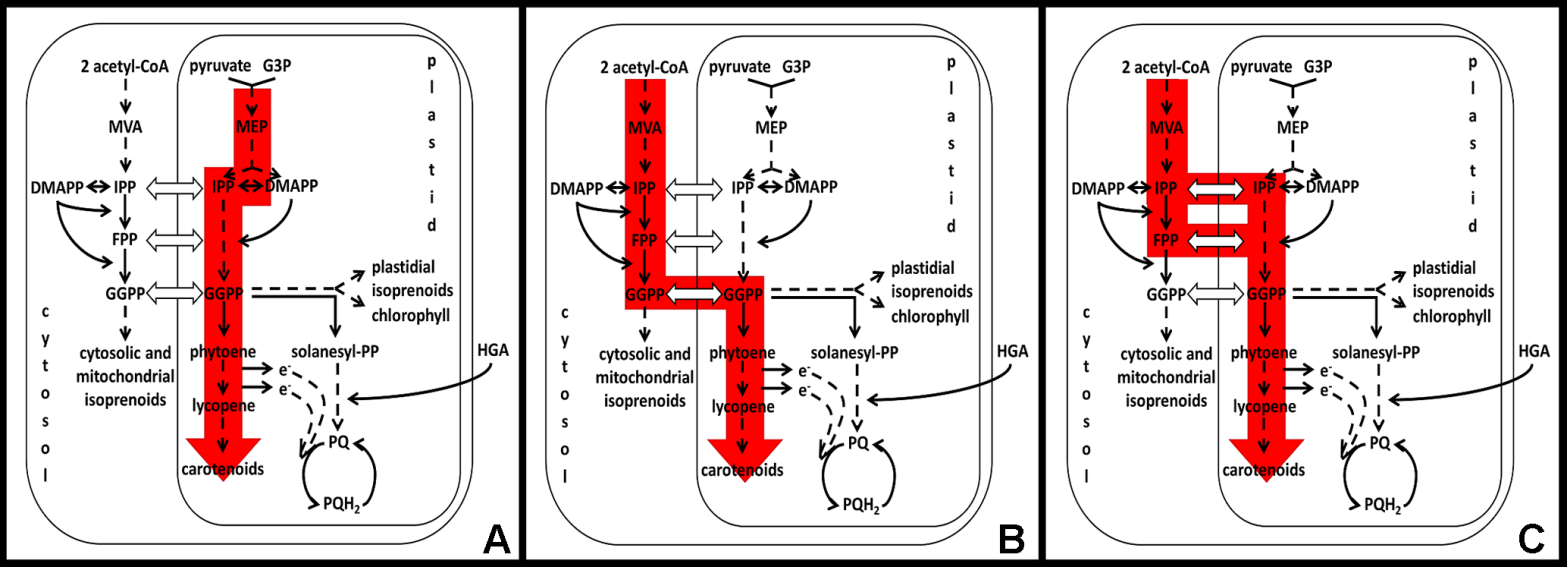
Independent pathways contributing towards carotenoid-dependent chloroplast biogenesis in Arabidopsis embryos. **(A)** MEP-dependent pathway, **(B**, **C)** MEP-independent or MVA-dependent pathway. **(B)** is deduced from the current experimental knowledge and from the indirect evidence that GGPPS11cyt is active in the cytosol *in planta* (Ruiz-Sola et al., 2016a) and is the main source of GGPP for carotenogenesis and chloroplast biogenesis (Ruiz-Sola et al., 2016a; Ruiz-Sola et al., 2016b). **(C)** Alternative model for MEP-independent/MVA-dependent pathway, which is based on the assumption that GGPPS11cyt is not active in the cytosol *in planta* or is not the main source of GGPP for carotenogenesis and chloroplast biogenesis. Abbreviations: MVA, mevalonate; DMAPP, dimethylallyl diphosphate; IPP, isopentenyl diphosphate; FPP, farnesyl diphosphate; GGPP, geranylgeranyl diphosphate; G3P-D, glyceraldehyde 3-phosphate; MEP, 2-C-methyl-D-erythritol 4-phosphate; PQ, plastoquinone; PQH_2_, plastoquinone reduced.

Development of chloroplasts from proplastids, at least in Arabidopsis embryos, requires viable carotenoid pathway. When carotenoid pathway is blocked, chloroplasts remain in the proplastid stage (Park et al., 2002, Gutiérrez-Nava et al., 2004, Tian et al., 2007, Qin et al., 2007; Dong et al., 2007; Chao et al., 2014). Although MEP pathway is the main source of GGPP for the carotenoid biosynthesis and as such for carotenoid-mediated chloroplast biogenesis (**Figure 2A**; Ruiz-Sola et al., 2016b; Sun et al., 2018), chloroplasts still develop in embryos of these mutants (Gutiérrez-Nava et al., 2004 and **Figure S2**). Chloroplast formation in MEP pathway mutants shows a specific pattern and is different from that observed in the wild-type Arabidopsis embryos (**Figure 1D**, c,f). In MEP pathway mutants, formation of chloroplasts is stronger in the embryonic root and hypocotyl, while weaker or absent in cotyledons (Gutiérrez-Nava et al., 2004 and **Figure S2**). In wild-type embryos, chloroplasts are formed overall, but higher chlorophyll abundance is seen in cotyledons (**Figure 1D**, c,f; Gutiérrez-Nava et al., 2004; Tejos et al., 2010).

As such, we can differentiate two independent pathways that contribute towards carotenoid-dependent chloroplast biogenesis in Arabidopsis embryos. One requires plastidial MEP pathway (**Figure 2A**) and the other is MEP pathway independent (**Figures 2B, C**). It was proposed originally by Gutiérrez-Nava et al. (2004) that the MEP-independent pathway is noncell autonomous, e.g., that intermediates required for the chloroplast biogenesis originate from the maternal tissues. Nowadays, we know from the analysis of isoprenoid pathway mutants that maternal tissues are not sufficient to provide enough intermediates or end products to complement MEP-independent biosynthesis of isoprenoids. Lack of GGPPS11 as a main source of GGPP for the isoprenoid biosynthesis results in developmental arrest at the heart stage of embryo development (Ruiz-Sola et al., 2016a; Ruiz-Sola et al., 2016b), mevalonate (MVA) pathway mutants are embryo or gametophyte lethal (Vranová et al., 2013), and lack of farnesyl diphosphate (FPP) biosynthesis in the cytosol of *fps1fps2* double mutant results in embryo lethality (Closa et al., 2010). Yet, the MVA pathway is likely providing IPP for plastidial isoprenoid biosynthesis when the MEP pathway is not functional (**Figure 2B**). Because the phenotype of mutants expressing GGPPS11 only in the cytosol resembles the phenotype of the MEP pathway mutants, it is likely that GGPPS11cyt is part of this MEP-independent pathway for carotenoid-dependent chloroplast biogenesis in Arabidopsis (**Figure 2B**). This model accepts the existence of the functional GGPPS11cyt protein in Arabidopsis and is supported by the evidence that *GGPPS11* locus can, in *A. thaliana*, produce shorter transcripts containing only second in-frame ATG codon and that this shorter GGPPS11 protein is active (**Figure 1**, Ruiz-Sola et al., 2016a). We have also found out that both the first and second ATG seem to be conserved in orthologs of GGPPS11 in 1135 Arabidopsis accessions, suggesting functional relevance for both methionins (http://1001proteomes.masc-proteomics.org/#At4g36810.1; 1001 Proteomes tool at 1001 Genomes website for non-synonymous SNPs detection).

Nevertheless, although protein translated from the *GGPPS11cyt* transcript is active in Arabidopsis when ectopically expressed or when solely expressed as a consequence of the T-DNA insertion in the targeting sequence of *GGPPS11* (**Figure 1**, Ruiz-Sola et al., 2016a), the GGPPS11cyt-GFP protein was never clearly detected in parallel to the GGPPS11-GFP protein, which localizes to plastids, when full-length GGPPS11 fused to the GFP was expressed in Arabidopsis (Beck et al., 2013, Ruiz-Sola et al., 2016a). Therefore, it cannot be excluded that GGPPS11cyt is not translated from the short transcripts produced by the wild-type *GGPPS11* locus and that IPP, DMAPP, or FPP is actually transported to plastids and there converted to GGPP by plastidial GGPPS11 (**Figure 2C**).

Additionally, even when GGPPS11cyt is expressed in Arabidopsis, it may be that it complements only the heart stage to seedling embryo development, but not chloroplast biogenesis. GGPPS2, another GGPPS that resides in plastids, can provide GGPP for the MEP-independent chloroplast biogenesis in Arabidopsis embryos. To find out whether GGPPS11cyt in the *ggpps11-4* GGPPS11cyt mutant is responsible for chloroplast biogenesis, GGPPS11cyt should be expressed in *ggpps11ggpps2* loss-of-function mutant background.

## Data Availability

All datasets for this study are included in the manuscript and the supplementary files.

## Author Contributions

EV planned and designed the research; JK, DK, and MC performed research; and EV and DK wrote the paper.

## Funding

This research was supported by SCIEX fellowship to JK and the grant VEGA 1/0926/17 from the Scientific grant agency of the Slovak Ministry of Education, Science, Research and Sport and of the Slovak Academy of Sciences.

## Conflict of Interest Statement

The authors declare that the research was conducted in the absence of any commercial or financial relationships that could be construed as a potential conflict of interest.
