# Supplementary material for: Mutant-Based Model of Two Independent Pathways for Carotenoid-Mediated Chloroplast Biogenesis in Arabidopsis Embryos
Source: Front Plant Sci. 2019 Aug 27;10:1034. doi: 10.3389/fpls.2019.01034 (PMC6718698; doi:10.3389/fpls.2019.01034)
Supplement: Supplementary file 1 [file DataSheet_1.pdf]

Supplementary Material

1 Supplementary Figures and Tables

1.1 Supplementary Figures

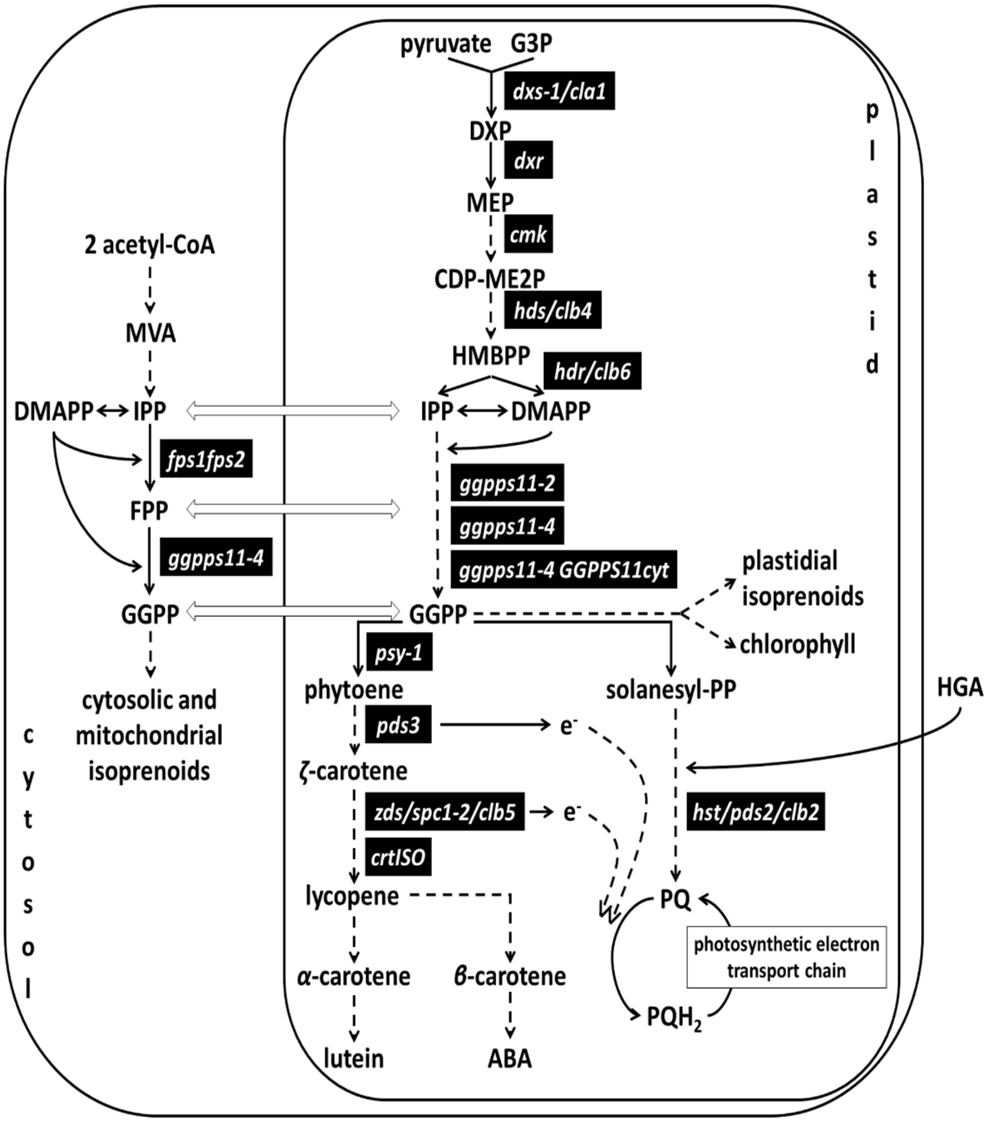

Supplementary Figure S1. Mutants mentioned or used in this work and their position in the isoprenoid pathway.

Pathway abbreviations: MVA- mevalonate, DMAPP- dimethylallyl diphosphate, IPP- isopentenyl diphosphate, FPP- farnesyl diphosphate, GGPP- geranylgeranyl diphosphate, G3P-D-glyceraldehyde 3-phosphate, DXP- 1-deoxy-D-xylulose 5-phosphate, MEP- 2-C-methyl-D-erythritol 4-phosphate, CDP- ME2P- 2-C-methyl-D-erythritol 2,4-cyclodiphosphate, HMBPP- 1-hydroxy-2-methyl-2-butenyl 4-diphosphate, PQ- plastoquinone, PQH_2- plastoquinone reduced

Mutant abbreviations, genes, lines and references:

all are loss-of-function mutants

fps1fps2	FPPS1FPPS2 (At4g17190, At5g47770) double mutant, Closa et al., 2010
ggpps11-4	GGPPS11 (At4g36810), Ruiz-Sola et al., 2016a,b
ggpps11-2	GGPPS11 (At4g36810), Ruiz-Sola et al., 2016a
ggpps11-4	GGPPS11cyt- GGPPS11 (At4g36810), this work
dxs-1/cla1	DXS1 (At4g15560), *cla1* this work
dxr	DXR - (At5g62790), SAIL_142_E06 this work
cmk	CMK (At2g26930), SALK_107310 this work
hds/clb4	HDS (At5g60600), *clb4* Gutiérrez-Nava et al., 2004
hdr/clb6	HDR (At4g34350), SALK_026807 this work, *clb6* Gutiérrez-Nava et al., 2004
psy-1	PSY (At5g17230), Ruiz-Sola et al., 2016a
pds3	PDS3 (At4g14210), Qin et al., 2007
zds/spc1-2/clb5	ZDS (At3g04870), *spc1-2* Dong et al., 2007, *clb5* Gutiérrez-Nava et al., 2004
crtISO	CRTISO (At1g06821), Park et al., 2002
hst/pds2/clb2	HST (At3g11945), *pds2* Tian et al., 2007, Chao et al., 2014, *clb2* Gutiérrez-Nava et al., 2004

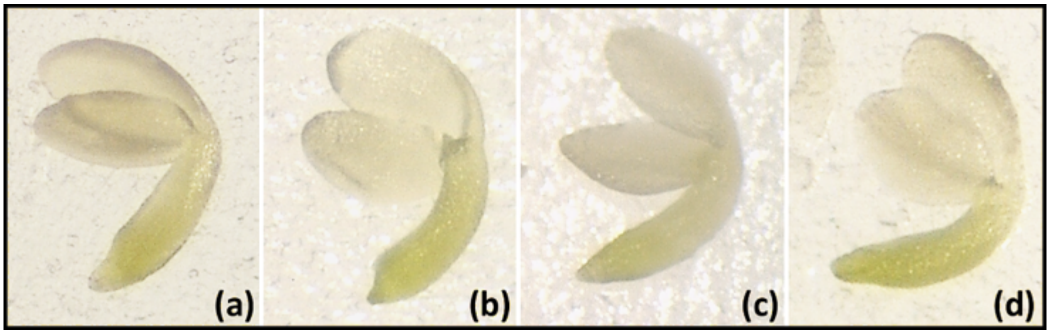

Supplementary Figure S2. Embryos mutated in the MEP pathway.

Phenotypes of embryos defective in DXS (a), DXR (b), CMK (c) and HDR (d). Embryos were analyzed visually under stereo microscope. Following mutant lines were used: *cla1* (a), SAIL_142_E06 (b), SALK_107310 (c) and SALK_026807 (d).

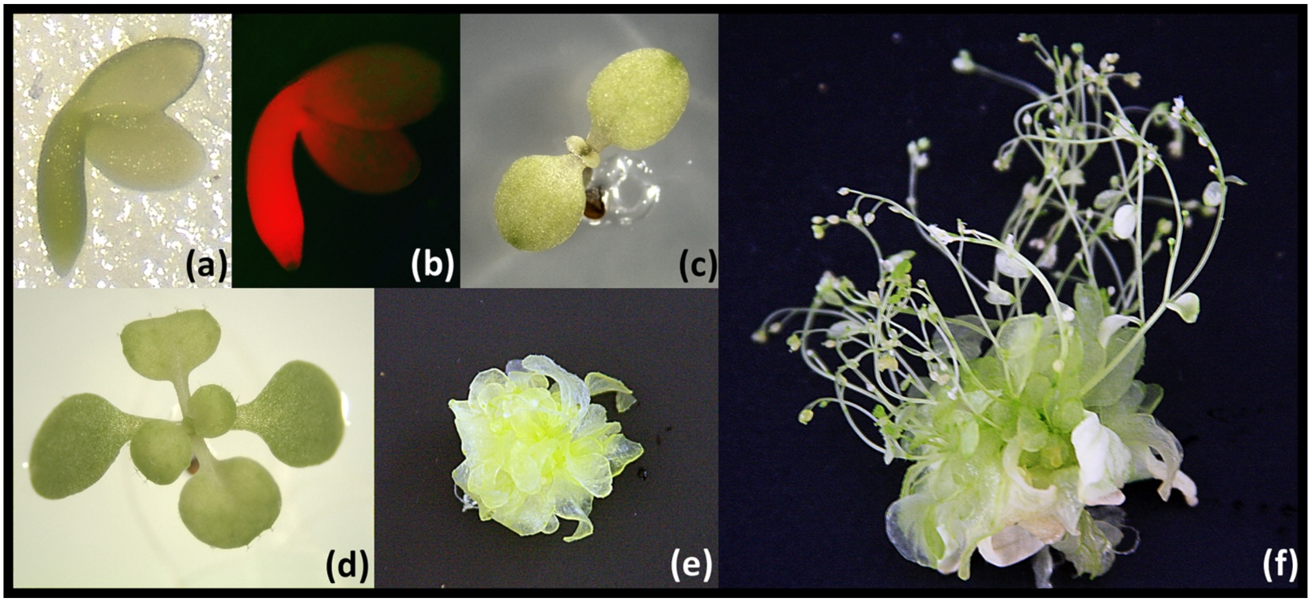

Supplementary Figure S3. Partial complementation of *ggpps11-4* phenotype by GGPPS11cyt.

Phenotypes of representative pale green/green embryo (a-b), 9-days old seedling (c), 17-days old plantlet (d) and 2-months old plants (e-f) that developed from these embryos; (a-f) *ggpps11-4* mutant complemented by GGPPS11cyt. Seedlings and plants were cultivated on MS plates supplemented with 1% sucrose, phosphinotricin (PPT) 10 µg/ml and kanamycin (Kan) 25 µg/ml. Embryos, seedlings and plantlets were all analyzed visually using stereo microscope (a, c, d) or fluorescence microscope (b) or camera (e, f).

1.2 Supplementary Tables

Primer name	*Sequence 5´→ 3´*
	CLONING
G11cyt_fwd	CACCATGACAAAAGAAGACAATCTACG
G11cyt_rev	TCAGTTCTGTCTATAGGCAATGTAATT
pG11_fwd	CCGCTCGAGGAAACTCACACCCACACAC
pG11_rev	GACTAGTGGCGATTTCTGAAATCTG
	GENOTYPING
G11wt_fwd	CAGTGACTCTAGGTTCATGGATTGTTGTTC
G11wt_rev	AATACTCAAAAAGACTGAAACCAACCAGGG
G11cyt_ins_fwd	TACAAAAAAGCAGGCTCC
G11cyt_ins_rev	TCAGTTCTGTCTATAGGCAATGTAATT
	SITE-DIRECTED MUTAGENESIS
G11cytNS_fwd	CTCTTTCGATTTCATCTCGTACATCATCACC
G11cytNS_rev	GGTGATGATGTACGAGATGAAATCGAAAGAG

Supplementary Table S1. Primer list

2 Supplementary Materials and Methods

Plant Materials and Growth Conditions

All *A. thaliana* (L.) Heynh lines in this study are in Columbia background. Plants heterozygous for the lethal *ggpps11-4* mutation (Ruiz-Sola *et al.*, 2016a) were grown from seeds of the T-DNA insertion line SAIL_712_D06 (Sessions *et al.*, 2002). Heterozygous MEP pathway loss-of-function mutants were grown from seeds of the following lines *dxs-1* (cla1), *dxr* (SAIL_142 E06), *cmk* (SALK_107 310), *hdr* (SALK_026 807). Seeds were obtained from the European Arabidopsis Stock Centre (http://arabidopsis.info/) or obtained as a gift from M. Rodríguez-Concepción (CRAG, Barcelona, Spain) and P. León (Universidad Nacional Autónoma de México, Mexico City, Mexico). Seeds were surface-sterilized and sown on Murashige and Skoog (MS) medium (Duchefa) containing 0,8% w/v plant agar (Duchefa), 1% w/v sucrose and 10 µg/ml phosphinotricin. Resistant seedlings were transferred from the plate to the soil to allow flowering. Both, *in vitro* and *ex vitro* plant cultures were cultivated in a climate-controlled growth chamber under 16/8 photoperiod, 22 °C and 60% RH.

Complementation of the *ggpps11-4* loss-of-function mutant

Cloning. *GGPPS11cyt* was amplified from cDNA from wild type *A. thaliana* with primers G11cyt_fwd and G11cyt_rev (TableS1). Proof-reading Pwo DNA polymerase (Roche) was used at 95 °C 3 min; 35x (95 °C 30 s, 50 °C 30 s, 68 °C 1 min 30 s); 68 °C 10 min. 943 bp amplicon of *GGPPS11cyt* was inserted into pENTR/D-TOPO vector (Invitrogen) and in subsequent Gateway® recombination reaction (Invitrogen) the insert was moved into the binary destination vector pK7WG2,0 (https://gateway.psb.ugent.be/search; Karimi *et al.*, 2002). The 35S promoter (p35S) of the expression vector pK7WG2,0_*GGPPS11cyt* was cut out with *Sal*I and *Spe*I enzymes (NEB) and native GGPPS11 promoter (*pG11*) was inserted instead. Prior to exchange, *pG11* was amplified from the wild type *A. thaliana* gDNA with a pair of primers pG11_fwd pG11_rev (Table S1) as 1361 bp long product. PCR cycling was performed with Pwo DNA polymerase (Roche) at 95 °C 3 min; 35x (95 °C 30 s, 52 °C 30 s, 68 °C 1 min 30 s); 68 °C 10 min followed by 15 min long incubation with Taq DNA polymerase (Eurogentec) to enable TA cloning. Amplified *pG11* sequence was cloned into pCR2.1 TOPO-TA vector (Invitrogen) and subsequently cut out as *Xho*I/*Spe*I (NEB) fragment, which was ligated into digested pK7WG2,0_*GGPPS11cyt* creating expression vector pK7WG2,0_pG11::*GGPPS11cyt*. All recombinant plasmids were propagated in *E. coli* TOP10 cells in LB (Difco) medium upon 50 µg/ml kanamycin (cloning vectors) or 100 µg/ml spectinomycin (expression vectors) selection pressure.

A. thaliana transformation. pK7WG2,0_pG11::*GGPPS11cyt* vector was transformed into *Agrobacterium tumefaciens* C58C1 (pMP90) (Koncz & Schell, 1986). Positive clones were selected on LB (Difco) plates based on 50 µg/ml rifampicin, 40 µg/ml gentamycin and 100 µg/ml spectinomycin resistance and were brought into floral-dip suspension following the protocol of Logemann *et al.* (2006). 6-8 weeks old plants heterozygous for *ggpps11-4* loss-of-function allele (Ruiz-Sola *et al.*, 2016b) were transformed and cultivated under previously described growth conditions until seed harvesting. Screening of primary transformants took place on selective MS medium (Duchefa) supplemented with 1% w/v sucrose, 10 µg/ml phosphinotricin and 25 µg/ml kanamycin.

Genotyping. The genotypes of primary *Arabidopsis* transformants as well as complemented *ggpps11-4* mutants were verified by PCR amplification preceded by DNA extraction. A leaf sample was snap-freezed in liquid nitrogen and grinded to a fine powder in the TissueLyser II (Qiagen). 300 µl of DNA extraction buffer containing 200 mM Tris-HCl pH7.5, 250 mM NaCl, 25 mM EDTA and 0.5% SDS was added. The sample was briefly vortexed and centrifuged for 8 min at 15000 rpm. The supernatant was transferred into a fresh tube and gently mixed with 350 µl isopropanol. The sample was incubated for 10 minutes at RT and centrifuged for 15 min at 15000 rpm. The supernatant was discarded and the pellet was dried in a vacuum concentrator prior to resuspension in 100-150 µl water of molecular biology grade. Extracted DNA served as template in PCR-based genotyping (Table S1). Two sequences were looked for in analyzed plants – wild type *GGPPS11* (G11wt) giving 1307 bp product and inserted *GGPPS11cyt* (G11cyt) giving 975 bp amplicon. Both reactions were performed with DreamTaq polymerase (Thermo Fisher Scientific) at 95 °C 5 min; 35x (95 °C 30 s, 54 °C 30 s, 72 °C 1 min 30 s); 72 °C 5 min.

Subcellular localization of GGPPS11cyt.

The gene construct encoding eGFP tagged GGPPS11cyt protein was introduced to the *Arabidopsis* wild type plants by *Agrobacterium* mediated transformation as described previously. Recombinant destination vector was derived from pK7FWG2,0 (https://gateway.psb.ugent.be/search; Karimi *et al.*, 2002) by Gateway® cloning technology using LR Clonase® II Enzyme Mix (Invitrogen) and entry vector (pENTR/D-TOPO, Invitrogen) harboring mutated form of *GGPPS11cyt* lacking the stop codon (*GGPPS11cytNS*). The stop codon was removed by site-directed mutagenesis of already prepared pENTR/D-TOPO_*GGPPS11cyt* according to Edelheit et al. (2009). Primers *G11cytNS_fwd* and *G11cytNS_rev* (Table S1) were used for the purpose. Plant expression of *GGPPS11cyt-GFP* was controlled by native *pGGPPS11* promoter, which replaced the original *p35S* of the pK7FWG2,0 vector in the aforementioned restriction-ligation reaction.

3 Supplementary References

Chao, Y., Kang, J., Zhang, T., Yang, Q., Gruber, M.Y., Sun, Y. (2014). Disruption of the homogentisate solanesyltransferase gene results in albino and dwarf phenotypes and root, trichome and stomata defects in *Arabidopsis thaliana*. *PLoS One* 9 (4):e94031. doi:10.1371/journal.pone.0094031

Closa, M., Vranová, E., Bortolotti, C., Bigler, L., Arró, M., Ferrer, A., Gruissem, W. (2010). The *Arabidopsis thaliana* FPP synthase isozymes have overlapping and specific functions in isoprenoid biosynthesis, and complete loss of FPP synthase activity causes early developmental arrest. *Plant J.* 63 (3):512-525. 10.1111/j.1365-313X.2010.04253.x.

Dong, H., Deng, Y., Mu, J., Lu, Q., Wang, Y., Xu, Y., Chu, C., Chong, K., Lu, C., Zuo, J. (2007). The Arabidopsis Spontaneous Cell Death1 gene, encoding a ζ-carotene desaturase essential for carotenoid biosynthesis, is involved in chloroplast development, photoprotection and retrograde signalling. *Cell. Res.* 17 (5):458-470. doi:10.1038/cr.2007.37

Edelheit, O., Hanukoglu, A., Hanukoglu, I. (2009). Simple and efficient site-directed mutagenesis using two single-primer reactions in parallel to generate mutants for protein structure-function studies. *BMC Biotechnol.* 9:61. doi:10.1186/1472-6750-9-61

Gutiérrez-Nava, M.d.l.L., Gillmor, C.S., Jimenez, L.F., Guevara-García, A., León, P. (2004). Chloroplast biogenesis genes act cell and noncell autonomously in early chloroplast development. *Plant Physiol.* 135 (1):471-482. doi:10.1104/pp.103.036996

Karimi, M., Inzé, D., Depicker, A. (2002). Gateway vectors for Agrobacterium-mediated plant transformation. *Trends Plant Sci.* 7 (5):193-195. doi:10.1016/S1360-1385(02)02251-3

Koncz, C., Shell, C. (1986). The promoter of Tl-DNA gene 5 controls the tissue-specific expression of chimeric genes carried by novel type of Agrobacterium binary vector. *Mol. Gen. Genet*. 204:383-396, doi: 10.1007/BF00331014

Logemann, E., Birkenbihl, R.P., Ulker, B., Somssich, I.E. (2006). An improved method for preparing Agrobacterium cells that simplifies the Arabidopsis transformation protocol. *Plant Methods* 2 (1):16. doi:10.1186/1746-4811-2-16

Park, H., Kreunen, S.S., Cuttriss, A.J., DellaPenna, D., Pogson, B.J. (2002). Identification of the Carotenoid Isomerase Provides Insight into Carotenoid Biosynthesis, Prolamellar Body Formation, and Photomorphogenesis. *Plant Cell* 14 (2):321-332. doi:10.1105/tpc.010302

Qin, G., Gu, H., Ma, L., Peng, Y., Deng, X.W., Chen, Z., Qu, L.J. (2007). Disruption of phytoene desaturase gene results in albino and dwarf phenotypes in Arabidopsis by impairing chlorophyll, carotenoid, and gibberellin biosynthesis. *Cell. Res.* 17 (5):471-482. doi:10.1038/cr.2007.40

Ruiz-Sola, M.A., Barja, M.V., Manzano, D., Llorente, B., Schipper, B., Beekwilder, J., Rodríguez-Concepción, M. (2016a). A Single Arabidopsis Gene Encodes Two Differentially Targeted Geranylgeranyl Diphosphate Synthase Isoforms. *Plant. Physiol.* 172 (3):1393-1402. doi:10.1104/pp.16.01392

Ruiz-Sola, M.A., Coman, D., Beck, G., Barja, M.V., Colinas, M., Graf, A., Welsch, R., Rütimann, P., Bühlmann, P., Bigler, L., Gruissem, W., Rodríguez-Concepción, M., Vranová, E. (2016b). Arabidopsis geranylgeranyl diphosphate synthase 11 is a hub isozyme required for the production of most photosynthesis-related isoprenoids. *New Phytol*. 209 (1):252-264. doi:10.1111/nph.13580

Sessions, A., Burke, E., Presting, G., Aux, G., McElver, J., Patton, D., Dietrich, B., Ho, P., Bacwaden, J., Ko, C., Clarke, J.D., Cotton, D., Bullis, D., Snell, J., Miguel, T., Hutchison, D., Kimmerly, B., Mitzel, T., Katagiri, F., Glazebrook, J., Law, M., Goff, S.A. (2002). A high-throughput Arabidopsis reverse genetics system. *Plant Cell* 14 (12):2985-2994. doi:10.1105/tpc.004630

Tian, L., DellaPenna, D., Dixon, R.A. (2007). The pds2 mutation is a lesion in the Arabidopsis homogentisate solanesyltransferase gene involved in plastoquinone biosynthesis. *Planta* 226 (4):1067-1073. doi:10.1007/s00425-007-0564-5
